# Sustainable Aquafeed Formulations Containing Insect Larval Meal and Grape Marc for the New Zealand Farmed Abalone

**DOI:** 10.1155/2023/8887768

**Published:** 2023-10-10

**Authors:** Natalia Bullon, Andrea C. Alfaro, Moganakumaar Manivannan, Seyedehsara Masoomi Dezfooli, Ali Seyfoddin

**Affiliations:** ^1^Drug Delivery Research Group, School of Science, Auckland University of Technology, Auckland, New Zealand; ^2^Aquaculture Biotechnology Research Group, School of Science, Auckland University of Technology, Auckland, New Zealand; ^3^The New Zealand Abalone Company, Bluff Invercargill, New Zealand

## Abstract

The aquaculture industry has been criticised for the excessive use of fish meal (FM) in feeds due to the utilisation of wild fish in the formulation and the exacerbation of overfishing marine resources. Land-based abalone aquaculture mainly uses commercial feeds (CFs) to promote faster growth, which include FM as a primary protein component. Alternative ingredients, such as insect meal (IM) and grape marc (GM) are potential candidates for FM replacement due to their suitable nutritional profile and sustainable production. This paper reports on a novel nutritional approach for the New Zealand farmed abalone, which replaces FM with IM by 10% and includes a waste by-product (GM) by 30% as a potential prebiotic source. The study was performed in two stages: (a) physico-chemical determination of diets delivered in an alginate matrix (experimental diets) and their stability in seawater compared to CF and (b) evaluation of growth and feed intake for the New Zealand black-foot abalone. There were significant differences between experimental diets and CF in terms of sinking rate, particle weight, and microscopic observations. Water stability of the experimental diets was increased by 50% in 24 and 48 hr compared to CF, producing less solid waste, and potentially reducing cleaning efforts in the farm. The inclusion of IM and GM did not compromise overall animal growth or their feed conversion ratio, however, further evaluation need to be explored in the future research. The findings revealed that the developed encapsulated feeds are a more stable food delivery method for *Haliotis iris* compared to the CF. Furthermore, both IM and GM can be included in feed formulations as a more sustainable strategy without compromising weight and shell gains in the abalone farming.

## 1. Introduction

Aquaculture is the fastest growing industry in the world in terms of protein supply, providing almost 50% of the total aquatic animal production [1]. In New Zealand, aquaculture is still a growing industry, exporting to 81 countries with annual sales of around NZD $650 million [2]. Although the New Zealand aquaculture industry is expected to grow five times by 2035, its current advancement has been hampered by the lack of knowledge of optimal nutritional profiles and effective feeding technologies to promote growth and reduce environmental impacts simultaneously [3]. One of the main contributors to the reduced sustainability of current aquafeeds is the use of fish meal (FM). The high price of FM is the result of its global scarcity and unsustainable future. Great efforts to replace FM in formulations with plant meals and concentrates have been investigated. However, the direct use of plant protein is still problematic due to the large cultivation area and water needed. Therefore, alternative ingredients, especially those derived from wastes from other productions are seen as potential candidates in the aquaculture feeds. Alternative ingredients for replacement, such as insect meals (IMs) and waste-by-products (e.g., grape marc (GM)) have been included in some aquafeeds to a certain extent but are often accompanied by a reduced feed quality due to poor digestibility of ingredients or lack of attractiveness to the species cultivated.

IMs have a good nutritional profile of protein, lipids, and carbohydrates [4] and other important nutrients that make them a good replacement for FM [5, 6]. Insect production is more sustainable than fish production because it uses less land area [7], produces less greenhouse gas [8], and less ammonia emissions [7], has an efficient feed conversion [9], and can be grown on organic waste-products from other industries.

GM, a by-product of the winery industry derived from *Vitis vinifera*, consists of the skins and seeds left after pressing. New Zealand has an abundant supply of this material, but its disposal has become problematic. GM possesses several nutritional qualities, including low levels of digestible carbohydrates, polyphenols (anthocyanins, catechins, flavonols, and phenolic acids), large amounts of resveratrol found in red grape skins and pulp, high levels of polyunsaturated fatty acids (over 60%), and more than 2% being omega-3 fatty acids [10]. These nutritional attributes make it promising for use in the aquaculture nutrition. However, the physical properties of GM need to be enhanced to improve water stability and nutrition retention. GM is primarily composed of polymers including 18.5% cellulose, 8.2% hemicellulose, and 56.4% lignin [11], giving it excellent adsorption capabilities [11]. Despite these qualities, the incorporation of GM in aquafeeds is very limited. To our knowledge, only GM derivatives have been used in feeds for *Haliotis laevigata* [12] with great success. The use of steam distilled GM (Acti-Meal®) has resulted in improved growth rates and feed conversion ratios (FCRs) for *Haliotis laevigata* when compared to commercial diets and feed without GM supplementation [12]. However, the direct use of GM in aquaculture feeding practices has not been previously explored.

Two of the most important concerns that come along with the development of feeds for aquaculture are the possible effects of alternative ingredients on the stability of the diet during seawater immersion and the potential pollution of the water column due to excessive phosphorus, carbon, and nitrogen [13]. Out of the solid wastes produced in aquaculture, uneaten feed residues are considered the biggest cost proportion in aquaculture settings [14]. Uneaten commercial feeds (CFs) normally disintegrate quickly, especially during abalone feeding activity, and such waste is collected when tanks are cleaned. Improvement of apparent digestibility of diet formulations, reduction of excessive leaching of nutrients (e.g., phosphorus, nitrogen, and carbon) and promoting faecal consistency constitute the first steps to improve diet formulations and feeding strategies to reduce solid waste excretion and improve farm water quality. Many types of IM can be included in aquafeeds, resulting in differences in terms of floatability, pellet duration, water absorption, and expansion ratio [15, 16]. The level of IM inclusion affects the fat content of the final feed, decreasing the pellet expansion and therefore affecting floatability [15]. Increased moisture in feeds increases pellet floatability due to increased starch gelatinisation, maintaining air bubbles inside the pellets [15].

Around 75% of the nitrogen (N) and phosphorus (P) from the feeds are usually unutilised and remain as waste in the culturing tanks [17]. Phosphorus is an essential element required by all fish for normal growth and may be included in excess to promote growth in the aquatic animals. This cultivation practice can cause excessive discharge of phosphorus into the environment, thus, increasing eutrophication with associated ecosystem impacts. Although some effluent P (such as faecal and uneaten P from feed) can be collected, soluble P is non-recoverable and is inevitably discharged into the environment without any existing regulation. Nitrogen waste is mostly associated with dissolved nitrogen in the form of amino acids released from the feeds and animal excretion of ammonia and faeces.

The inclusion of new ingredients into aquafeeds requires evaluation of feed physical characteristics, such as floatability, matrix erosion, texture profile analysis, and nutritional composition, and chemical characteristics, such as carbon, nitrogen, and phosphorus components, which are retained or released once the feed is placed in the water. These evaluations may provide insight on the physico-chemical and nutritional potential of alternative ingredients, such as IM and GM when included in abalone feed formulations.

The aim of this study was to develop innovative encapsulated feeds, formulated with alternative ingredients (IM and GM) for abalone aquaculture. The study provides a comprehensive characterisation of physical and chemical properties of the various experimental diets, as well as an evaluation of animal feed intake and growth. It is envisioned that this work will provide the foundation for future aquafeed formulations with a wider range of supplements optimised for different species and farm requirements.

## 2. Materials and Methods

### 2.1. Diet Preparation

Four experimental diets were formulated to contain graded levels of FM, corn meal (CM), IM, and dried GM (Table 1). The CF Marifeed® (Hermanus, South Africa) was provided by the New Zealand Abalone Company and used as the control feed.

Experimental diets were prepared by initially mixing pre-weighed, ground ingredients in a commercial blender (Nutri Bullet 600 household mixer). All ingredients were mixed except starch. Starch syrup at 10% was prepared and sequentially added to the dry mixture to create a dough with a consistent texture. The dough was spread out on a flat tray and dried at a temperature of 65°C for 16 hr. Subsequently, the dried dough was pulverised using a commercial blender (Nutri Bullet 600 household mixer) for 30 s and the resulting powder was sifted twice through a kitchen sieve (approx. 40 mesh number).

### 2.2. Encapsulation of Experimental Diets

Encapsulation was performed according to Masoomi et al. [18] with small modifications using a calcium chloride and alginate solution. Alginate solution 1% *w*/*v* was mixed with the feed powder. Encapsulation took place after placing the mixture feed powder-alginate in a calcium chloride solution (0.1 M). Formed beads were washed with 100–200 mL of distilled water and then arranged on an oven tray covered lined with aluminium foil. These beads were left to air dry inside a commercial oven (Piron PF8906, Italy), maintaining a temperature of 65°C for 16 hr. After that, dried beads were carefully sealed in a vacuum bag to prevent potential contamination. Proximate analyses were conducted on all the diets in triplicate.

### 2.3. Proximate Analyses of Feeds

Proximate compositional analyses, including crude protein, crude lipid, ash, and moisture content were carried out on both the experimental diets and the CF following AOAC guidelines [19]. The moisture content was determined in three replicates by utilising a convection oven at 135°C for 3 hr. To prevent denaturation or oxidation of metabolites, the experimental diets and the CF were ground up into powder using a grinder (IKA A11 model analytical mill, Germany) with the addition of liquid nitrogen.

#### 2.3.1. Protein Analyses

The nitrogen levels of the experimental diets and the CF were assessed using a CE-440 Elemental Analyser (Exeter, Chelmsford, Massachusetts, USA). The process involved combustion and reduction temperatures at 980 and 700°C, respectively, with pure oxygen as the combustion gas and pure helium as the carrier gas. The purge and combustion times were 15 and 20 s, respectively. To determine the protein content, the nitrogen value was multiplied by the conversion factor 6.25.

#### 2.3.2. Lipid Analyses

For lipid determination in the diets, the extraction of crude fat extraction was carried out following the method described by Bligh and Dyer [20] for small samples. In summary, 0.1 g of the dried and ground experimental diets or CF were mixed with 1 mL chloroform and 2 mL methanol, and 800 *µ*L distilled water. The mixture was then vortexed for 2 min, after which 1 mL chloroform was added. The solution was vortexed again for 30 s, and then 1 mL distilled water was added to create separate layers. The mixture was vortexed again for 30 s and subsequently centrifuged at 3,000 rpm for 5 min. The lower organic solvent layer was carefully collected into a pre-weighed 8 mL glass tube and evaporated using a nitrogen stream. Finally, the net weight of the lipids in the sample was recorded.

#### 2.3.3. Ash Analyses

A sample of 0.5 g from each dried ground experimental diets and CF were placed in a furnace for combustion at 550°C for 6 hr. Ashes were weighed, and the difference expressed as ash percentage (%) in the total sample.

#### 2.3.4. Carbohydrate Analyses

Carbohydrate contents were assessed using the Anthrone method [21]. In brief, 30 mg of the ground samples were digested with 2.5 N HCl for 3 hr. Following this, the resulting homogenate was centrifuged at 15,000 rpm for 15 min at a temperature of 4°C. The supernatant was collected and diluted 50 times for a better determination. Each tube containing 1 mL sample (diluted if needed for better determination) had 4 mL of Anthrone reagent added to it. These tubes were sealed and incubated at 100°C for 15 min. After cooling, the developed colour was measured against a glucose standard and a blank at 620 nm in a UV—visible spectrophotometer equipped with a quartz cuvette. The results were expressed in milligram of glucose/g sample.

#### 2.3.5. Fibre Analysis

Total dietary fibre was determined in experimental diets and CF using Megazyme kit (Catalogue K-TDFR-100A/K-TDFR-200A 04/1, Megazyme, Ireland). In brief, 1 g of ground samples were hydrolysed subsequently with *α*-amylase, protease, amyloglucosidase, and further precipitated with ethanol to produce a residue that was used for protein and ash determination.

### 2.4. Physical Characterisation of Feeds

#### 2.4.1. Particle Size and Weight of Feeds

Experimental beads for diets F, FI, FG, FIG and CF were measured (*n* = 50) in the longest axis using a calliper Mitutoyo (0.01 mm). Weight was measured using a digital balance (Sartorious CPA2P).

#### 2.4.2. Sinking Rate of Feeds in Seawater

The sinking rate of particles (*n* = 25) was measured by placing individual beads or pellets in a measuring cylinder filled with seawater. The height of the seawater column was 16 cm. The time that a particle travelled the column height from top to bottom was recorded as sinking time and divided by 16 cm.

#### 2.4.3. Water Absorption Index (WAI) and Water Solubility Index (WSI) of Feeds

WAI measures the volume occupied by the granules after swelling in excess of water. WSI indicates the percentage of polysaccharides released from the granule after the addition of excess water. WAIs for the experimental diets and CF were determined by the method outlined by Anderson [22]. The ground diets (0.85 g) were suspended in deionised water (10 mL) in a tared 50 mL centrifuge tube. The suspension was stirred constantly for 3 min (Vortex genie 2, Scientific industries, USA) and centrifuged at 5,000 rpm for 10 min. The supernatant was decanted into a tarred aluminium cup and dried at 120°C for 2 hr. The cup and its contents were cooled in a desiccator and reweighed on a sensitive weighing scale, and the difference in weight (W_SS_) was obtained. The mass of the gel remaining in the centrifuge tube (*W*_*g*_) was also obtained. The WAI and WSI were calculated using Equations (1) and (2).

(1) $$\begin{array}{c} \text{WAI}=\left(\frac{W_{g}}{W_{\text{ds}}}\right), \end{array}$$

where *W*_*g*_ is the weight of gel (g) and *W*_ds_ is the weight of dry sample (g).

(2) $$\begin{array}{c} \text{WSI}\left(\%\right)=\left(\frac{W_{\text{ss}}}{W_{\text{ds}}}\right)\times 100, \end{array}$$

where *W*_ss_ is the weight of dry solids of supernatant (g) and *W*_ds_ is the weight of dry sample (g).

#### 2.4.4. Matrix Erosion of Feeds

This experiment was conducted in farm conditions (Bluff, Invercargill, New Zealand) in tanks without abalone to evaluate the percentage of solids disintegrated from the capsule or pellet after 24 and 48 hr of immersion in seawater. Three mesh cloth bags (200-*µ*m nylon mesh filter) containing 2.5 g of experimental diets or CF were placed at the bottom of a tank containing 90 L of filtered seawater (100-*µ*m nylon mesh filter). A flow-through water system was maintained at a rate of 1.5 L/min, which equates to a total water exchange of 40 times per day. Water temperature was maintained between 16.1 and 19°C and dissolved oxygen 92.7%– 99.6%. After 24 and 48 hr the bags were removed and dried at 50°C for 16 hr and stored for further analysis.

The dried weights of particles were measured and the percentage of matrix erosion in the particles was calculated as per Equation (3):

(3) $$\begin{array}{c} \text{Matrix erosion}\left(\%\frac{w}{w}\right)=\left(\frac{W0-W}{W0}\right)\times 100, \end{array}$$

where *W*0 is the initial dry weight of the beads and *W* is the mass of dry beads after incubation in seawater.

#### 2.4.5. Microscopy of Feeds

The morphology and microstructure of the experimental diets and CF were examined using a scanning electron microscope (SEM) (Hitachi SU-70, Japan). For SEM imaging, samples were dehydrated at 70°C (experimental diets) and randomly selected. The dried diets were placed on double sided adhesive carbon tapes on aluminium stubs and then coated with a thin layer of platinum under vacuum for 60 s by an ion sputter coater (Hitachi E-1045, Japan). The elemental analysis was performed using an energy dispersive spectrometer (EDS). Samples were observed under SEM at 15 kv and EDS spectra were obtained to screen the elemental composition of the beads using Noran System 7 (NSS) microanalysis system software (Thermoscientific, USA).

#### 2.4.6. Texture Profile Analysis of Feeds

Texture analysis was performed using a Stable Micro Systems Texture Analyser equipped with a Film Support Rig (HDP/FSR) on a Heavy-Duty Platform (HDP/90) with a 5-mm stainless steel probe (P/55) and a 5-kg load cell. The texture analyser was set to measure force in the compression mode with a pre-test speed of 2.0 mm/s, a test speed of 0.5 mm/s, and a post-test speed of 2.0 mm/s, strain 1%, trigger force 1 g, and a time of 5 s for diets in time 0 and 16 hr, and a pre-test speed of 2.0 mm/s, a test speed of 0.5 mm/s, and a post-test speed of 2.0 mm/s, strain 50%, trigger force 1 g and a time of 5 s for diets in time 24 and 48 hr. Target mode was set to a distance of 5 mm. Data acquisition rate was set at 500 pps. Hardness, gumminess, and chewiness data were collected for statistical analyses. Hardness is described as the force required to compress a material by a given amount, gumminess as the energy required to break down a semi-solid ready for swallowing and chewiness as the energy required to chew a solid food into a state ready for swallowing (all of them with unit N—newtons) [23].

#### 2.4.7. Durability Index

The durability index is a measure of the strength of pellets/capsules to resist mechanical handling during transportation, storage, and subsequent use. The durability index was determined as outlined by Irungu et al. [15] with modifications. About 15 ± 0.1 g of each dried sample was sifted on a series of seven sieves placed in this order: 2, 1, 0.6, 0.425, 0.25, 0.15, 0.075 mm. The samples were placed at the highest pore sieve 2 mm (*Wi*) and the series of sieves placed in a flask mounted on a Lab-Line shaker, which was shaken for 20 min. The beads were reweighed (*Wr*) in each sieve and the pellet durability index (PDI) was calculated using the Equation (4) as follows:

(4) $$\begin{array}{c} \text{PDI}\left(\%\right)=\left(\frac{Wr}{Wi}\right)\times 100. \end{array}$$

### 2.5. Chemical Characterisation of Feeds

These determinations were conducted in two different settings. The first setting included the evaluation of the nutrient leaching (amino acids, phosphorus, carbon, and nitrogen levels) from the uneaten feeds in tanks without abalone at 0, 24 and 48 hr. These data were useful to understand the nutrient leaching/adsorption in uneaten feed when abalone excretion waste was not present in the tanks. The second setting included the evaluation of nutrient leaching/adsorption (phosphorus, carbon, and nitrogen levels) from the uneaten feed in tanks with abalone at 0, 24, 48, and 96 hr as per Section 2.6. These data were used to evaluate if abalone excretion waste has a role to play in the nutrient leaching/adsorption of the uneaten feeds. In both cases, the uneaten feed was collected, dried for 20 hr at 60°C in a convection oven and then stored until further analysis.

#### 2.5.1. Amino Acid Level of Feeds

The procedure for extracting total amino acids followed the methodology outlined by Paramás et al. [24] and detailed previously by Bullon et al. [25]. In brief, three replicates of 50 mg dried samples of diets were hydrolysed with 1.5 mL 12 M concentrate hydrochloric acid in a heating block at 110°C for 22 hr. The solution was filtered through filter paper and adjusted to pH 4–6 with NaOH 1 M. Once neutralised, samples were centrifuged at 3000 rpm for 5 mins and supernatants were kept frozen until analysis. After extraction, samples were derivatised using the AccQ-Tag (6-aminoquinolyl-*N*-hydroxysuccinimidyl carbamate) method for liquid chromatography–mass spectrometry (LC–MS) as detailed by Bullon et al. [25]. The amino acid standard used contained 37 amino acids (A9906 amino acid standard Sigma 485845-1G) and D-4 alanine was used as an internal standard. LC–MS system using an Agilent 1260 Infinity Quaternary LC System (Santa Clara, CA 95051 USA) was used for determination with a column Phenomenex Kinetex evo C18 (2.1 × 150 mm, 1.7 *µ*m). The mobile phase was composed of water containing 0.1% (*v*/*v*) formic acid (A) and acetonitrile containing 0.1% (*v*/*v*) formic acid (B) with an initial gradient condition of 99 : 1 (A : B). The total run time for each sample was 30 min.

#### 2.5.2. Phosphorus Level in Feeds

Microwave plasma atomic emission spectroscopy (MP-AES) was used to determine phosphorus in experimental diets and CF at different times of seawater immersion. Dried samples (0.5 g) were digested with 10 mL of 65% nitric acid in a microwave at 195°C over a period of 20 min and at 180°C for 20 min. Samples were filtered using a plastic funnel with filter paper (541 Whatman filter paper) and 50 mL 2% nitric acid, and further diluted 25 times to be measured against a calibration curve with diammonium hydrogen orthophosphate (NH_4_)_2_HPO_4_.

#### 2.5.3. Carbon and Nitrogen Level of Feeds

Total carbon and nitrogen concentrations were measured in experimental diets and CF at different times of seawater immersion. Ground samples were weighed at 2–4 mg in a tin capsule. Nitrogen and carbon detections were determined by a CE-440 Elemental Analyser (Exeter, Chelmsford, Massachusetts, USA). The combustion and reduction temperatures were 980 and 700°C, respectively, with pure oxygen as the combustion gas and pure helium as the carrier gas. The purge and combustion times were 15 and 20 s, respectively. Samples were measured in three replicates and acetonitrile was used as a standard.

### 2.6. Experimental Animals and Tank Systems

**Figure 1:**
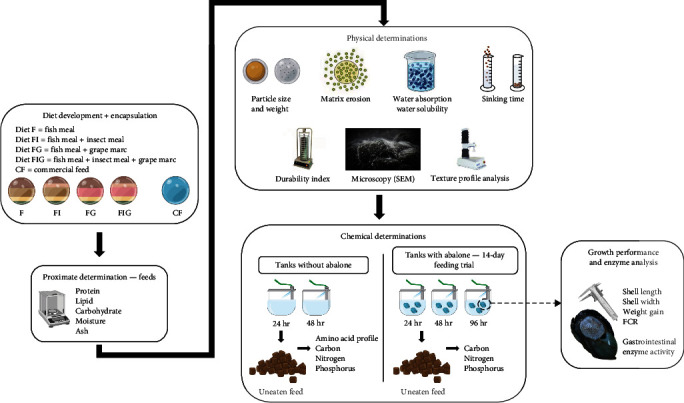
Summary of the experimental design and the experimental workflow.

Juvenile abalone (*Haliotis iris*) (*n* = 45) with an initial mean weight of 5.7 ± 0.8 g, mean shell length of 34.9 ± 1.6 mm, and shell width of 23.5 ± 1.1 mm were provided by the New Zealand Abalone Company (Bluff, Invercargill, New Zealand) and transported to the facilities of the Auckland University of Technology (Auckland, New Zealand) overnight in December 2022 (Figure 1). Upon arrival, the animals were immediately placed in seawater with an ambient temperature of 14°C. Abalone were acclimated for 2 days and then kept for 14 days for the feeding trial. During this time, water parameters, such as pH, nitrate, nitrite, ammonia, salinity, and temperature were measured before and after water exchanges every day. An electronic thermometer was used for temperature measurement and was calibrated according to manufacturer instructions. A liquid saltwater master kit (Api Fishcare Inc.) was used for pH, nitrite, nitrate, and ammonia measurements and was calibrated against standard solutions provided by the manufacturer. Salinity was measured with a salinity refractometer and calibrated with a standard solution provided by the manufacturer.

Three tanks containing 5 L of seawater were allocated per treatment. Each tank contained three abalone. Tanks were aerated and maintained in a photoperiod of 24 hr of darkness unless feedin*g* and cleaning activities were performed. Four experimental diets (F: fish meal; FI: fish meal + insect meal; FG: fish meal + grape marc, and FIG: fish meal + insect meal + grape marc) and a CF were used in the experiment. Food was provided daily in excess (0.5 g per tank) at 15:00 hr after cleaning. Tanks were cleaned daily, and uneaten feed was collected in a 1-mm mesh. Uneaten feed was dried for 20 hr at 60°C in a convection oven and then stored at −80°C until further analysis.

Apart from the feed provided, a cloth bag (200-*µ*m nylon mesh filter) containing 1.2 g of dried experimental diets or CF was placed on the surface of the tank. Those feeds were used to measure how the uneaten feed was affected in terms of phosphorus, carbon, and nitrogen levels over time, and the impact of the seawater as it was modified by the abalone's physiological activities (e.g., respiration, faeces production). After 24, 48, and 96 hr, the bags with feed were removed from the tanks, the residue was removed and dried for 20 hr at 60°C in a convection oven and then stored until further analysis of carbon, nitrogen, and phosphorus content could be performed (Sections 2.5.2 and 2.5.3).

At the end of the 14-days feeding trial, all animals were measured and dissected, and their tissues collected for digestive enzyme analysis (Section 2.6.2).

#### 2.6.1. Growth Performance

Growth measurements were recorded at the start and at the end of the 14 day-feeding trial. Removal of abalone from tanks was performed with the aid of a blunt knife by carefully lifting the foot off the surface of the tank. Then, abalone were dried with paper towels, and their maximum shell lengths and widths (mm) and total abalone wet weights (g) were recorded. Lengths were measured with a vernier calliper (Mitutoyo 0–125 mm, Warwickshire, UK) to the nearest 0.1 mm, and weights were measured with a digital balance to the nearest 0.1 g. The following measurements were calculated:

Average daily weight gain (AD_w_) as in Equation (5)

(5) $$\begin{array}{c} {\text{AD}}_{w}=\frac{T_{\text{fw}}-T_{\text{iw}}}{14\text{ days}}, \end{array}$$

where T_fw_ is total weight at the end of the trial and T_iw_ is total weight at the beginning of the trial.

Average daily shell length gain (AD_SL_) as in Equation (6)

(6) $$\begin{array}{c} {\text{AD}}_{\text{SL}}=\frac{{\text{SL}}_{f}-{\text{SL}}_{i}}{14\text{ days}}, \end{array}$$

where SL_f_ is the shell length (mm) at the end of the trial and SL_i_ is the shell length (mm) at the beginning of the trial.

Average daily shell width gain (AD_SW_) as in Equation (7)

(7) $$\begin{array}{c} {\text{AD}}_{\text{SW}}=\frac{{\text{SW}}_{f}-{\text{SW}}_{i}}{14\text{ days}}, \end{array}$$

where SW_f_ is the shell width (mm) at the end of the trial and SW_i_ is the shell width (mm) at the beginning of the trial.

FCR as in Equation (8)

(8) $$\begin{array}{c} \text{FCR}=\frac{\text{feed consumed}}{\text{abalone weight gain}}, \end{array}$$

where FCR and the feed consumed is the difference of the feed given and the uneaten feed.

#### 2.6.2. Enzyme Analysis

Abalone shells were removed before dissection. The gastrointestinal region (combined tissue as no clear distinction was possible) was separated from the adductor muscle. The samples were immediately snap-frozen in liquid nitrogen and stored at −80°C prior to the analysis of digestive enzyme activity.

Before analysis, the samples were thawed, and 0.7–0.8 g of tissue was weighed and homogenised in 5 mL of MiliQ water. A homogeniser (IKA Ultraturrax T25, Germany) was used at 17,000 rpm for 60 s. Samples were then centrifuged at 12,000 rpm for 10 min at 4°C. The supernatant was removed completely and saved for the different determinations of enzymes which were performed at different pH.

The supernatants were analysed for trypsin, *α*-amylase, and lipase activity using spectrophotometric techniques and commercial enzyme test kits. Trypsin was determined at 405 nm at 3 and 60 min (Catalogue AB102531; Abcam, Australia) in a kinetic mode. Amylase was determined at 405 nm at 0 and 3 min due to the high activity of the samples (Catalogue AB102523; Abcam, Australia) in a kinetic mode. Lipase was determined at 412 nm at 0 and 30 min (Catalogue AB102525; Abcam, Australia) in a kinetic mode. Total protein was determined using a Bicinchoninic Acid (BCA) Protein Assay Kit with bovine serum albumin solution as the standard (Catalogue 23225; Thermo Fisher Scientific, USA). Specific enzyme activities were defined as the amount of enzyme that catalysed the conversion of 1 *µ*mol of substrate per minute per milligram of protein (i.e., U mg soluble protein^−1^).

## 3. Statistical Analyses

One-way analysis of variance was carried out to detect the effect of the diet formulation on the physical characteristics, proximate composition, growth measurements, and abalone gastrointestinal enzyme activity when a normal distribution was found (Kolmogorov–Smirnov test, *p* < 0.05) followed by pairwise comparisons with Tukey's post hoc test. Homogeneity of variances was analysed using Levene's test when samples followed a normal distribution (*p* < 0.05). Two-way analysis of variance was carried out to detect the influence of the type of feed and time of immersion in seawater on the amino acid profile, phosphorus, carbon, and nitrogen levels. Significant differences between readings of the different samples were evaluated using the statistical software XLSTAT 2022.3.1 (Addinsoft, New York, USA) with Tukey's post hoc comparison tests, where statistical significances were found.

## 4. Results

### 4.1. Physical Characterisation of the Feeds

The particle weight of experimental diets ranged depending on the ingredients (Table 2). Diet F (4.5 ± 1.2 mg) and FI (4.1 ± 1.3 mg), which did not contain GM, were significantly lower in weight compared to diet FG (10.3 ± 4.2 mg) and FIG (7.9 ± 3.1 mg). Regarding particle size, diet F (2.1 ± 0.5 mm) and FI (2.4 ± 0.7 mm) were significantly smaller in size compared to diet FG (4.4 ± 1.7 mm) and FIG (3.9 ± 1.2 mm) and CF (3.9 ± 0.3 mm). In terms of sinking rate, dried experimental beads performed better than fresh beads and frozen beads better than dried beads. In the frozen form, diets containing GM had significantly higher sinking rates compared to the diets without GM. The matrix erosion results showed that all experimental diets had significantly higher seawater stability compared to the CF in 24 and 48 hr. The diets containing GM had significantly higher WAIs compared to the diets not containing GM. The WSI indicated that diet FG had lower water solubility compared to other diets, and diet FI and the CF had the highest solubility with 9.3 ± 0.1% and 9.9 ± 0.2%, respectively.

#### 4.1.1. Microscopy of the Feeds

**Figure 2:**
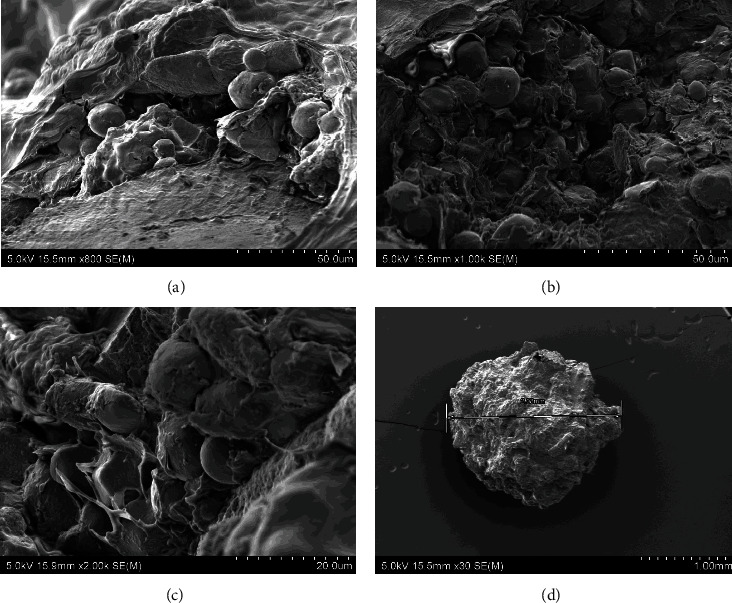
The SEM photographs of diet F: (a) diet F dried at 35°C for 16 hr, (b) diet F dried at 50°C for 16 hr, (c) diet F dried at 70°C for 16 hr, and (d) entire photo of alginate bead from diet F. Abbreviation: diet F (fish meal based).

**Figure 3:**
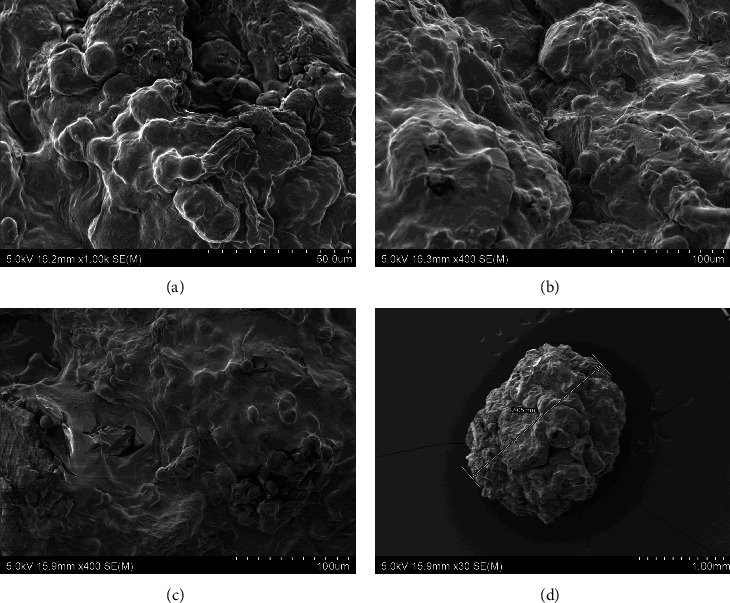
The SEM photographs of diet FI: (a) diet FI dried at 35°C for 16 hr, (b) diet FI dried at 50°C for 16 hr, (c) diet FI dried at 70°C for 16 hr, and (d) entire photo of alginate bead from diet FI. Abbreviation: diet FI (fish meal + insect meal).

**Figure 4:**
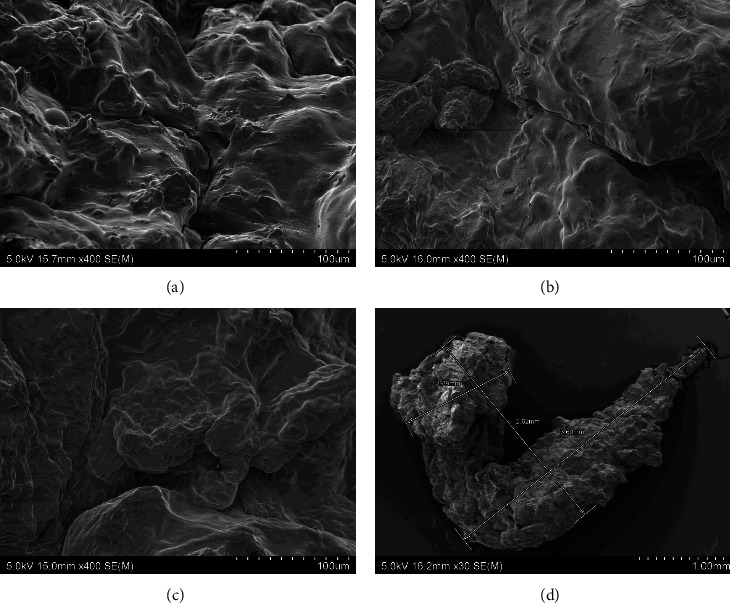
The SEM photographs of diet FG: (a) diet FG dried at 35°C for 16 hr, (b) diet FG dried at 50°C for 16 hr, (c) diet FG dried at 70°C for 16 hr, and (d) entire photo of alginate bead from diet FG. Abbreviation: diet FG (fish meal + grape marc).

**Figure 5:**
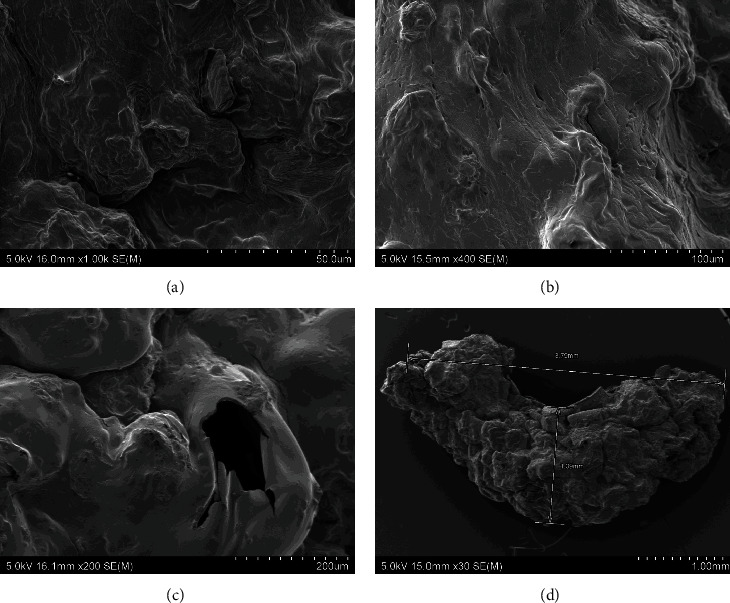
The SEM photographs of diet FIG: (a) diet FIG dried at 35°C for 16 hr, (b) Diet FIG dried at 50°C for 16 hr, (c) diet FIG dried at 70°C for 16 hr, and (d) entire photo of alginate bead from diet FIG. Abbreviation: diet FIG (fish meal + insect meal + grape marc).

**Figure 6:**
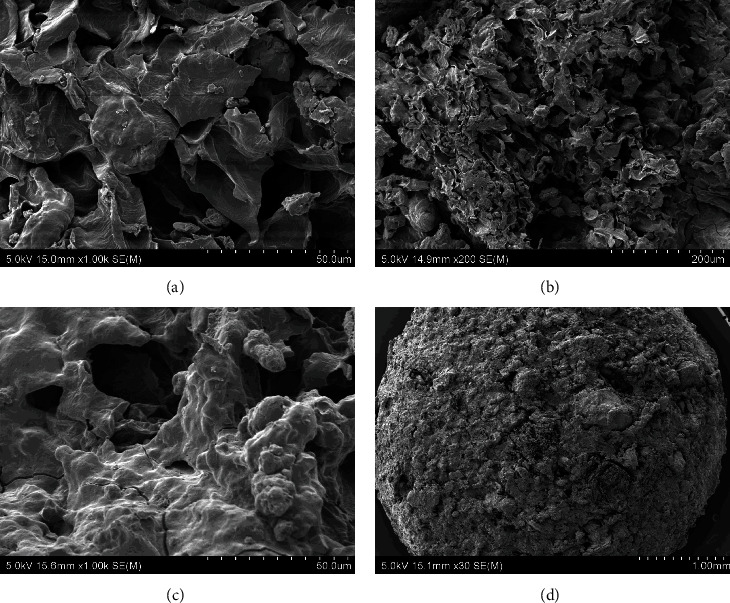
The SEM photographs of commercial feed (CF). Abbreviation: commercial feed (CF).

The scanning electron microscopic images of the experimental diets showed that the alginate beads were formed by three-dimensional porous structures consisting of a high number of connected irregular sheets with some grain-shaped bulges on the surfaces. There was no presence of scaffolds, typical in alginate hydrogels. The surface of the four experimental diets was even, without many breakages in different drying temperature (35, 50, and 70°C). This consistency in bead surfaces exposed to different temperatures suggest that the drying temperature does not affect the porous structures of the beads. The morphology of the experimental diets was well-compacted without observable significant changes in the porous structures which indicate the absence of effect of GM or IM inclusion in the morphology of the dried beads. However, the shape of the beads was different between diets with GM and without GM. Diet F (Figure 2) and FI (Figure 3) were generally rounded compared to diet FG (Figure 4) and FIG (Figure 5), which were elongated. Comparably, the CF showed a more porous structure the surface suggesting more water absorbance (Figure 6).

#### 4.1.2. Texture Profile Analysis (TPA)

**Figure 7:**
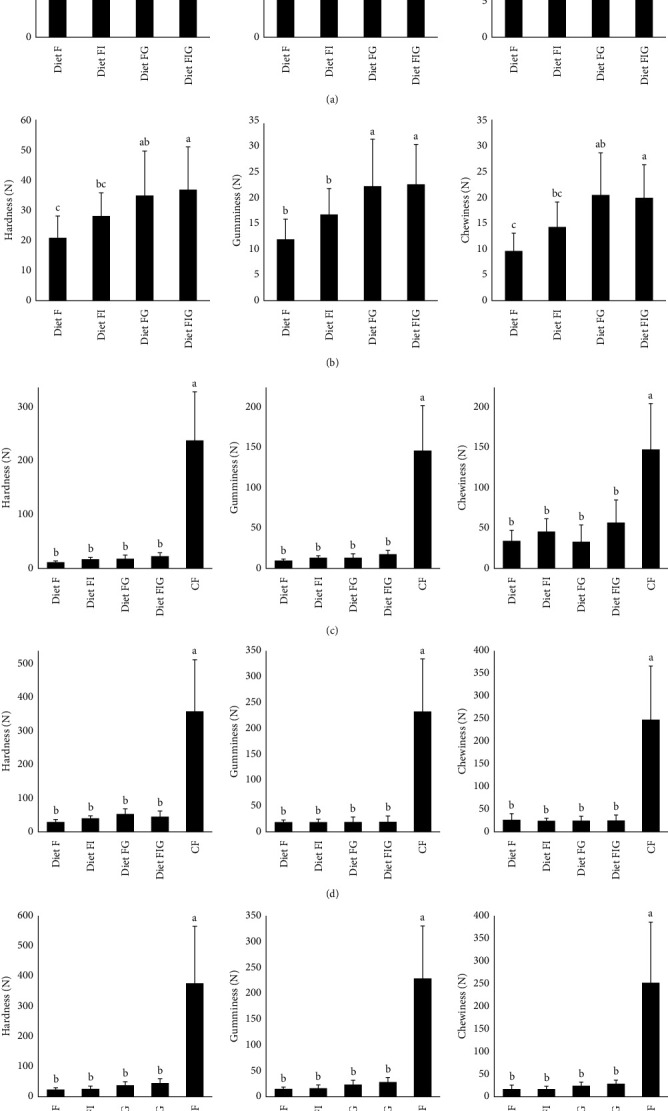
Texture profile analysis of fresh experimental diets and commercial feed at different storage conditions (fresh and refrigerated) (a, b) and at different time points of immersion in seawater (16, 24, and 48 hr) (c–e). Data are presented as means ± SD (*n* = 25). Bars with the same superscript (a, b, c) are not significantly different from Tukey post hoc tests (*p* < 0.05). Abbreviations: Diet F (fish meal based), FI (fish meal + insect meal), FG (fish meal + grape marc), FIG (fish meal + insect meal + grape marc), and commercial feed (CF).

Overall, the TPA of the experimental diets and CF showed that the time of immersion in seawater affects the hardness, gumminess, and chewiness of the diets. When experimental diets were fresh, the inclusion of IM or GM resulted in significantly higher gumminess and the inclusion of IM significantly increased the hardness of the baseline diet F (Figure 7(a)). When experimental beads were refrigerated (2–8°C), diets that included GM resulted in significantly higher hardness, gumminess, and chewiness compared to the diets without GM (diet F) (Figure 7(b)). Compared to fresh beads, gumminess increased almost twice during refrigerated conditions compared to diet F and FI. After 16 hr in seawater, the experimental diets resulted in significantly lower hardness, gumminess, and chewiness compared to the CF, but there were no significant differences among the experimental diets (Figure 7(c)). After 24 hr in seawater, the CF showed almost 10 times more hardness, gumminess, and chewiness compared to the experimental diets (Figure 7(d)). There were no significant changes among the experimental diets F, FI, FG, and FIG. After 48 hr in seawater, the values of hardness, gumminess, and chewiness were not significantly different among experimental diets but were significantly different between commercial diet and experimental diets (Figure 7(e)). Hardness, gumminess, and chewiness of the CF remained quite similar between 24 and 48 hr.

#### 4.1.3. Durability Index

The durability index showed the ability of experimental diets and CF to withstand mechanical handling. The results showed that after 20 min of shaking, most of the beads from diet F and FI (no GM) were less than 2 mm but more than 1 mm in size with 62.48% and 71.06%, respectively (Table 3). The diet FI was the most affected among all the experimental feeds during shaking with 2.35% of solids less than 1 mm but more than 0.6 mm in size. Contrarily, diets with GM (FG and FIG) showed better durability compared to diets without GM with 43.22% and 59.03% of beads remaining in the 2-mm sieve, respectively. All the experimental diets had at least 97% of particles bigger than 1 mm after 20 min of shaking indicating that the inclusion of IM and GM did not significantly influence the durability of the beads. CF had 99.92% pellets bigger than 2 mm and the effect of abrasion was minimum.

### 4.2. Proximate Analyses of Feeds

Proximate composition results of the experimental diets are shown in Table 4. The estimated energy content was in the range of 18.1–20.4 KJ/g for all experimental diets. The energy content of the CF was not disclosed by the manufacturer but was calculated according to the kilojoules assigned for protein, carbohydrate, and lipid proportion (Table 4 legend 3). Experimental diets containing GM (FG and FIG) contained more energy compared to other experimental diets (F and FI) considering the carbohydrate levels resulted from subtraction (Table 4 legend 1). Although this calculation is still used in food design to calculate energy from nutrients, values should be used with caution as GM contains high levels of non-digestible carbohydrates rather than digestible carbohydrates. All experimental diets had the same level of protein and the CF had significantly higher levels of protein. Experimental diets containing GM (FG and FIG) contained significantly lower levels of reducing sugars with 8.9 ± 1.9% and 9.1 ± 1.1%, and significantly higher dietary fibre levels with 16.1 ± 0.1% and 15.4 ± 0.3%, respectively. The CF had the lowest level of dietary fibre (3.7 ± 0.3%). Diets containing insect meal (FI and FIG) had significantly higher levels of lipid compared to the diets without IM. The lowest lipid level was found in CF (1.2 ± 0.3%). Diet FG had significantly higher levels of ash (14.5 ± 0.3%) compared to the other experimental diets and CF had the lowest level (6.8 ± 0.4%). The CF had the highest level of moisture (10.7 ± 0.1%) and diet FI the lowest (3%).

### 4.3. Chemical Characterisation of the Feeds

#### 4.3.1. Amino Acid Level of Feeds

The amino acid profiles of the experimental diets and the CF at time 0, 24, and 48 hr on seawater are shown in Table 5. Amino acids L-histidine, L-arginine, and L-lysine were the only essential amino acids that were significantly different among diets. Experimental diets FI and FIG resulted in significantly higher levels of L-histidine compared to CF. All experimental diets had significantly higher levels of L-arginine and L-lysine compared to CF. Effects of time immersed in seawater showed that time significantly increased L-histidine, L-arginine in the first 24 hr of immersion, while significantly reduced taurine, L-threonine, L-valine, L-lysine, L-phenylalanine, and L-tryptophan. The interaction diet ^*∗*^ time showed that the levels of hydroxylysine and lysine varied significantly dependent on the type and time of immersion in seawater.

#### 4.3.2. Carbon, Nitrogen, and Phosphorus Levels in Feeds

For both experiments, diets with both, fish meal and insect meal (FI and FIG) resulted in less amount of phosphorus compared to diets that included fish meal only (F and FG) at time 0 hr. Time did not play a significant role in variations of P levels in the tanks without abalone (Table 6). However, time played a significant role in variations of P levels in tanks with abalone (Table 7). In tanks with and without abalone, P levels were significantly affected by the interaction of diet and time in seawater indicating an additive effect.

The two-way ANOVA showed that diet was the most critical factor that affects the C : N ratio in both tanks with (Table 7) and without abalone (Table 6). Similarly, the time of immersion in seawater played a significant role in the C : N ratio of diets in tanks with and without abalone. The interaction of diet and time of immersion in seawater played a significant role in both P levels and C : N ratio, in tanks without abalone, while it did not have a significant role in the C : N ratio in diets trialled in the tanks with abalone.

Diets with inclusion of either IM of GM produced higher C : N ratio at time 0 hr. Diet F had the lowest value at time 0 hr, indicating more N in the formulation. The two-way ANOVA also showed that diet is the most critical factor that affects P and C : N levels of uneaten feed compared to time of immersion. These levels are found to be significantly higher in the uneaten feed when abalone is present in the tanks, particularly the P levels which were almost duplicated. Both, P levels and C : N ratio of uneaten feed occur independently of abalone presence.

### 4.4. Feed Intake and Enzyme Activity

Due to the length of the feeding trial (14 days), evaluation of abalone growth was limited. However, the acceptance and feed intake were assessed to evaluate experimental diets. Results showed no significant differences in FCR among experimental diets and CF (Table 8). In terms of enzyme activity, there was a significant difference between diet FI and FG and FG and CF in trypsin. There was significant increase of amylase activity in diets which contained GM FG and FIG compared to the other experimental diets which did not contain GM. There were no significant differences among experimental diets and CF in the lipase activity.

## 5. Discussion

### 5.1. Physical Characterisation

Our study shows the potential of encapsulation for the delivery of nutrition and the inclusion of two alternative ingredients for the New Zealand abalone. The physical characteristics of the experimental diets suggest that these diets perform better compared to the CF. The particle size of the experimental diets was not significantly different from the CF. This finding suggest that the experimental diets were suitable for ingestion for juvenile abalone. The particle weight showed that the CF had the heaviest particles. The diets that included GM (FG and FIG) were heavier compared to diets without GM and this is supported by the values from the sinking rate test. Diets containing GM had significantly longer sinking rates than diets without GM, indicating that GM provides a certain “structural” characteristic that creates more density in the particle, yet particles do not sink faster. A possible explanation of this phenomenon is the formation of a complex lignin–alginate formed inside the particle. Lignin is an insoluble polymer highly present in GM and can create a higher stable polymer complex with calcium alginate matrix, which has a high capacity of water absorption [27]. This absorbent capacity is supported by the values of WAI. In fact, GM has been used in industrial applications to adsorb pigments from the aqueous solutions [11]. Although the experimental diets in their fresh and dried form had longer sinking rates than the CF, this was improved when they were frozen. This might indicate that the frozen delivery is the most adequate due to the bottom feeding nature of abalone and the risk of feeds being washed away. The frozen form might provide additional advantages, such as the delivery of probiotics in the gastrointestinal tract of abalone [28] promoting more viable bacteria compared to room temperature formulations [29, 30].

Our study shows that our experimental diets were more stable in seawater, less resistant to abrasion compared to CF and yet promoting growth in abalone. The solid loss in the CF was significantly higher compared to the experimental diets in both 24 and 48 hr. These results support the fact that the encapsulation method is more efficient at keeping the particles inside the capsule compared to extruded pellets. In fact, alginate beads are recognised for their water stability due to the calcium crosslinker, which provides a semi-permeable bead allowing the containment of the particles during prolonged times [31]. The CF showed lower stability in seawater due to the higher solubility (WSI), a higher porous structure (microscopy), and the higher density of powder reflected in the high weight of the CF compared to the experimental diets. Each pellet of CF of 3.9 ± 0.3 mm size contained 16.6 ± 2.3 g of powder, almost double that of the weight of the experimental diet beads. The durability index showed that the experimental diets with GM were more resistant to the mechanical handling than the diets without GM. Particles with high durability form fewer small particles during bagging, storage, and finally, show low degradation in pneumatic feeding devices when fed to aquatic animals [32, 33]. The CF has a completely different physical structure than the experimental diets, as evident from the microscopic analysis. The CF showed more predominant scaffolds and more chains extended through the surface, suggesting a more absorbing capacity as supported by the WAI and the WSI.

Another finding in our study was the non-significant variation of the texture profile of our experimental diets as time went on. Experimental diets that included IM and GM were more stable in seawater after 24 and 48 hr. However, their hardness, gumminess, and chewiness were not significantly different after 16, 24, and 48 hr of immersion in seawater. This observation is of particular interest as water stability does not jeopardise texture particle characteristics for abalone ingestion. Interestingly, the main variations among experimental diets occurred when they were in the fresh or frozen form. In the fresh form, the inclusion of GM and/or IM produced diets with significantly higher gumminess compared to the diets without GM and/or IM, but hardness nor chewiness were affected. In the frozen form, these traits were amplified showing that the inclusion of GM resulted in diets with significantly higher hardness, gumminess, and chewiness compared to the diets without GM. Conversely, the inclusion of IM did not cause significant variations. The texture analysis from the encapsulated diets with GM showed higher hardness, gumminess, and chewiness, particularly in the frozen form. This fact reinforces about the existence of the highly stable “lignin–calcium alginate matrix” in the experimental beads [27], which may trap water and ions from the water column. This matrix may resemble the “egg box model” cross-linking matrix present in the alginate beads [34]. Although quite stable, this structure is debilitated while immersion in seawater extends due to calcium sequestration from the hydrogel [35]. This structure is created by L-guluronate and D-mannuronate residues and provides support along with calcium via cross-linking. Once this structure is formed, the substances inside the “box” are slowly released from the gel depending on the divalent ions on the surroundings and pH. The rapid diffusion of small molecules through the alginate membrane [36] allows more particles and water to be absorbed. Due to these properties and the high ability of calcium alginate beads to “protect” molecules from the harsh environments of the stomach [37], they are of high interest for inclusion of symbiotics, probiotics, and post-biotics.

### 5.2. Chemical Characterisation

The proximate composition of the diets suggests that the inclusion of IM and GM affected the lipid, carbohydrate, dietary fibre, and ash composition. Diets containing only FM as a protein source (F and FG) were relatively higher in protein compared to the diets which included a mixture of fish meal and insect meal (FI and FIG), although the changes were not significant. These variations are caused by the reduced levels of protein of the insect *Tenebrio molitor* (around 50%) [38] compared to FM (around 53%) (data not shown in this study). In addition, IM contains a higher lipid proportion [38] compared to FM, reflected in the significantly higher lipid proportion of diets that included IM FI (7.0 ± 0.6%) and FIG (7.2 ± 0.3%). The most remarkable difference among experimental diets was in dietary fibre. Diets FG and FIG which contained GM, resulted in significantly lower levels of digestible carbohydrates (reducing sugars) and higher levels of dietary fibre. The GM non-digestible proportion is made of complex carbohydrates, such as oligosaccharides (OS) [39], which positively influence gut health by acting as prebiotics [40]. Usually, the beneficial effect of GM has been attributed mainly to polyphenols and antioxidants such as tocopherol. However, recent research has showed that oligosaccharides from grape seeds have a significant prebiotic activity on *Lactobacillus acidophilus* [41].

Our results suggest that the ingredients in the diet formulation affected the phosphorus level and C:N ratio over time. Diets that contained significantly higher levels of protein (F, FG, and CF) had lower C : N ratios. Diets that contained IM as a supplement and therefore, significantly lower levels of protein, had lower levels of C : N ratios. This finding supports the inclusion of IM to reduce the nitrogen load while still promoting growth of abalone. Therefore, the inclusion of IM would reduce the over-enrichment of N of the water column compared to FM [42]. Although the inclusion of IM successfully increased the C : N ratio, there are no data on the preferred C : N ratio for abalone feeds to promote growth. Most of the feeds used in aquaculture have a low C : N ratio of around 7–10 : 1 [43, 44], which implies low levels of C and high levels of N as it is the main element for protein formation.

The increased levels of P and C : N ratio in both tanks with and without abalone, showed that these levels are significantly affected by the diet composition rather than the time of immersion in seawater (0, 24, and 48 hr). In addition, the P levels, and the C : N ratio are strongly affected by the presence of abalone in the tanks, contributing to higher levels of P and N in uneaten feed as time went on. This finding suggests that abalone excretion plays a role in the C and N levels of uneaten feed [45, 46] where possibly dissolved nitrogen is absorbed in the capsule/pellet. As uneaten feed waste is partially discharged with the effluent water in farms [47], it corroborates the potential environmental pollution from enriched N-uneaten feed waste.

The amino acid profile of diets showed that the essential amino acids L-histidine, L-arginine, and L-lysine were mainly affected by the type of diet. From these amino acids, L-lysine is the most important as it is considered the first limiting amino acid in abalone [48, 49]. The findings in this study suggest that the experimental diets would have provided L-lysine equally or more than to what CF provided at least in the first 24 hr. Although the lysine levels for the CF at time 0 hr were the lowest, they promoted good growth over time. The L-lysine values decreased significantly in the first 24 hr for all diets supporting the fact that lysine supplementation might be needed to improve growth performance [50]. Another interesting finding was the significantly increased levels of arginine in the experimental diets which included IM and/or GM compared to the CF and diet F (only FM as a protein source) at time 0 hr. Along with lysine, arginine has been considered a limiting amino acid in abalone feeds [51]. Interestingly, our study showed that arginine was found to significantly increase in the first 24 hr along with L-histidine, L-methionine, and L-leucine. The increase of amino acids in the uneaten feed can be explained by the adsorbent capacity of the pellets/capsules according to their morphology and nutrient composition. The encapsulated diets may possibly adsorb compounds from the water column such as peptides and free amino acids from dead cell material [52, 53]. The capacity of the encapsulated diets to adsorb methionine, arginine, and histidine after 48 hr can be utilised for waste removal. The adsorbent capacity along with the improved seawater stability of encapsulated diets (only 7%–8% disintegration after 48 hr) facilitates uneaten feed waste removal with a sieve of 1 mm.

The essential amino acids taurine, L-threonine, L-valine, L-phenylalanine, and L-tryptophan were significantly reduced after 24 hr of immersion in seawater in all diets. Among these amino acids, taurine was the most reduced with almost 80% reduction. For this reason, some commercial formulations add taurine. Taurine is a recognised feed enhancer along with methionine or glutamine to promote faster growth [54, 55].

### 5.3. Growth Performance and Enzyme Activity

Our results from the 14-day feeding trial provided limited information for growth evaluation. Daily growth for abalone is around 0.1 mm, therefore, providing only a total of 1.4 cm approximately for comparison. A longer feeding trial is recommended to expect more significant differences among dietary treatments considering providing a settling period with the new introduced diets to avoid biases. However, growth data are presented here as a reference point for future research. Weight gain and growth indicated that the experimental diets were accepted and promoted growth in abalone which was one of the outcomes of this research. There was a high variability in diet FIG weight gains, suggesting that a bigger sample size of abalone should be considered in the future. High variability of weight and shell length is expected particularly in juvenile populations [56]. The FCR results suggest that the feed consumption of the experimental diets, in particular diet FG and FIG, were quite variable. This phenomenon might be explained by the gradual adaptation of abalone to GM. The FCR was not significantly different among the experimental diets and CF.

Our results corroborate that the digestive enzyme activity of abalone is modulated by the feed consumed. Previous studies have shown that enzyme adaptation takes place depending on the diet consumed in *Haliotis laevigata* [57] and *Haliotis fulgens* [58]. The presence of lignin in the diets with GM might have promoted a more stable polymer, yet not highly digestible. The high content of dietary fibre in diets that included GM might have caused abalone to spend more energy in digesting the fibre, thus, slowing down the allocation of energy for growth. As a result, the shell length was slightly reduced compared to diet F (only FM as protein source) and the CF. In addition, the increased activity of amylase in animals fed diets with GM supports the prebiotic nature of GM which is similar to other feed supplements such as probiotics, increases enzyme activity [59], therefore possibly increasing food absorption and efficiency [60].

## 6. Conclusions

The inclusion of alternative ingredients into aquatic feeds requires a holistic evaluation of many factors attributed to the feed, animal, and environment. In this study, we focused on the physical and chemical characteristics, feed delivery form, nutritional value, and feed intake of experimental diets that included IM and GM as a supplement. To our knowledge, this is the first study to formulate abalone feeds in an alginate bead form and compare performance with a CF in terms of physico-chemical characteristics and abalone growth. The experimental diets delivered in alginate beads were more stable in seawater over 24 and 48 hr compared to the CF. The inclusion of GM resulted in significantly harder particles when they were dried, thus increasing the capacity to resist abrasion during longer periods of storage and manipulation. However, the inclusion of GM and a combination of IM and GM resulted in frozen particles significantly harder, gummier, and chewier compared to diets without IM and GM. Interestingly, hardness, chewiness, and gumminess were reduced significantly in the experimental diets with GM as time went on. Our results indicate that the appropriate delivery for the experimental diets is frozen beads, which reduce the sinking rate and add formulation advantages such as the inclusion of bioactives or thermolabile compounds. Due to less solid loss after 24 and 48 hr, alginate beads may improve efficiency in farms allowing less cleaning which can be reduced from 48 to 96 hr. The growth data need further examination as there were no significant differences among treatments in 14 days. However, experimental diets were consumed by abalone. The inclusion of GM and the combination of GM and IM significantly increased amylase activity but did not increase trypsin and lipase activity compared to the diet without GM and IM and the commercial diet. Overall, the present study showed two viable alternative ingredients, IM and GM for abalone feeds that if introduced at 10% and 30%, promoted growth, feed intake and modulation in digestive enzymes. Longer studies are required to evaluate the long-term effect of both alternative ingredients along with the inclusion of micronutrients, such as vitamins and minerals for further feed optimisation.

## Figures and Tables

**Table 1:** Percentage (dry weight basis) composition of the experimental diets (g/100 g).

Ingredients (g/100 g diet)	Diet			
	F	FI	FG	FIG
Fish meal	35	25	35	25
Insect meal	–	10	–	10
Corn meal	30	30	–	–
Grape marc	–	–	30	30
Seaweed (dry) *Macrocystis pyrifera*	4	4	4	4
Starch (native maize flour)	10	10	10	10

*Note*: Fish meal was donated by Sandford NZ (New Zealand), maize starch was supplied by New Zealand starch (New Zealand Starch Ltd.), seaweed (*Macrocystis pyrifera*) was obtained from Southern Clams, grape marc was supplied by Bragato Research Institute and insect meal was purchased from Shanxian Yongzheng Pet food Co., Ltd. (Beijing, China). Corn meal was purchased from a local market. For encapsulation: sodium alginate was purchased from AcrosOrganics (Beijing, China) and calcium chloride (CaCl_2_) was purchased from Ajax Finechem (NSW, Australia). All the other chemicals used in this study were of analytical grades. Abbreviations: F, fish meal; FI, fish meal + insect meal; FG, fish meal + grape marc; FIG, fish meal + insect meal + grape marc.

**Table 2:** Physical characterisation of experimental diets and commercial feed (CF).

Physical parameters		Diet				
		F	FI	FG	FIG	CF
Particle weight (mg)	–	4.5 ± 1.2^d^	4.1 ± 1.3^d^	10.3 ± 4.2^b^	7.9 ± 3.1^c^	16.6 ± 2.3^a^
Particle size (mm)		2.1 ± 0.5^b^	2.4 ± 0.7^b^	4.4 ± 1.7^a^	3.9 ± 1.2^a^	3.9 ± 0.3^a^
Sinking rate (cm/s)	Fresh beads	0.46 ± 0.12^bc^	0.39 ± 0.05^c^	0.52 ± 0.09^b^	0.69 ± 0.30^a^	NA
	Frozen beads	0.23 ± 0.04^b^	0.22 ± 0.03^b^	0.30 ± 0.05^a^	0.33 ± 0.05^a^	NA
	Dried beads	0.25 ± 0.03^c^	0.27 ± 0.04^c^	0.57 ± 0.29^b^	0.75 ± 0.40^a^	0.16 ± 0.02^c^
Matrix erosion (%)	24 hr	4.6 ± 1.6^b^	0.1 ± 0.01^c^	0.5 ± 0.8^c^	0.7 ± 0.4^c^	13.9 ± 0.9^a^
	48 hr	13.3 ± 1.1^ab^	7.2 ± 2.3^c^	8.5 ± 0.7^bc^	7.3 ± 1.6^c^	17.5 ± 3.1^a^
WAI (g)	–	3.3 ± 0.2^c^	3.3 ± 0.1^c^	4.4 ± 0.2^a^	4.0 ± 0.05^b^	3.9 ± 0.04^b^
WSI (%)	–	8.0 ± 0.4^b^	9.3 ± 0.1^a^	5.1 ± 0.1^c^	7.6 ± 0.3^b^	9.9 ± 0.2^a^

*Note*: Data are represented by means ± standard deviation. For each parameter, significant differences are shown by different superscripts (Tukey's test, *p* < 0.05). Abbreviations: Diet F (fish meal based), FI (fish meal + insect meal), FG (fish meal + grape marc), FIG (fish meal + insect meal + grape marc), and commercial feed (CF). Particle weight and size (*n* = 50), sinking rate (*n* = 25), matrix erosion (*n* = 3), WAI, and WSI (*n* = 3). Sinking water performed in seawater temperature 22°C. WAI (g) water absorption index. WSI (%) water solubility index. NA = not applicable.

**Table 3:** Durability index of experimental diets and commercial feed according to pore size of sieves (mm).

Sieve (mm)	Diet				
	F	FI	FG	FIG	CF
2	36.08^de^	26.27^e^	43.22^d^	59.03^c^	99.92^a^
1	62.48^bc^	71.06^b^	56.02^c^	40.66^d^	0.02^f^
0.6	0.91^f^	2.35^f^	0.59^f^	0.29^f^	0.03^f^
0.425	0.16^f^	0.14^f^	0.05^f^	0.02^f^	0.00^f^
0.25	0.12^f^	0.11^f^	0.02^f^	0.00^f^	0.01^f^
0.15	0.09^f^	0.04^f^	0.02^f^	0.00^f^	0.01^f^
0.075	0.09	0.01^f^	0.07^f^	0.00^f^	0.01^f^

*Note*: Values are expressed in grams. Data represent means and standard deviation (*n* = 3). Means with the same superscript (a, b, c) in each column are not significantly different from Tukey post hoc tests (*p* < 0.05). Abbreviations: Diet F (fish meal based), FI (fish meal + insect meal), FG (fish meal + grape marc), FIG (fish meal + insect meal + grape marc), and commercial feed (CF).

**Table 4:** Proximate composition of experimental feeds and commercial feed.

Proximate composition	Diet				
	F	FI	FG	FIG	Commercial feed^4^
Protein (%)	30.4 ± 0.1^ab^	27.3 ± 0.3^b^	30.8 ± 0.7^ab^	26.4 ± 2.0^b^	32.4 ± 2.7^a^
Carbohydrate (%)^1^	47.9	50.8	45.4	49.3	46.3
Carbohydrate-reducing sugars (%)^2^	28.9 ± 1.3^b^	32.0 ± 3.1^ab^	8.9 ± 1.9^c^	9.1 ± 1.1^c^	39.1 ± 4.7^a^
Total dietary fibre (%)	9.0 ± 0.2^b^	7.5 ± 0.1^c^	16.1 ± 0.1^a^	15.4 ± 0.3^a^	3.7 ± 0.3^d^
Lipid (%)	4.0 ± 0.2^c^	7.0 ± 0.6^a^	5.3 ± 0.5^b^	7.2 ± 0.3^a^	1.2 ± 0.3^d^
Ash (%)	13.5 ± 0.1^b^	11.9 ± 0.1^c^	14.5 ± 0.3^a^	12.9 ± 0.3^b^	6.8 ± 0.4^d^
Moisture (%)	4.2 ± 0.01^b^	3.0 ± 0.01^b^	3.9 ± 0.03^b^	4.2 ± 0.8^b^	10.7 ± 0.1^a^
Energy (KJ per g)^3^	18.4	20.4	18.9	20.2	15.9

*Note*: Data represent means and standard deviation of three technical replicates. For each parameter, significant differences are shown by different superscripts (Tukey's test, *p* < 0.05). ^1^Carbohydrate proportion was calculated by difference 100 – (moisture + protein + lipid + ash). ^2^Carbohydrate was determined using reducing sugar method Anthrone. ^3^Total energy was calculated based on the physiological values at 23.4 kJ g^−1^ protein, 39.7 kJ g^−1^ lipid, and 17.2 kJ g^−1^ carbohydrates [26]. ^4^Commercial feed used was Marifeed S34. Abbreviations: Diet F (fish meal based), FI (fish meal + insect meal), FG (fish meal + grape marc), FIG (fish meal + insect meal + grape marc), and commercial feed (CF).

**Table 5:** Amino acid composition of experimental diets and commercial feed after immersion in seawater for 0, 24, and 48 hr.

Amino acid (mg/g sample)	Diet																	
	F		FI			FG			FIG			CF			ANOVA (*p* value)			
Time in seawater (hr)	0 hr	24 hr	48 hr	0 hr	24 hr	48 hr	0 hr	24 hr	48 hr	0 hr	24 hr	48 hr	0 hr	24 hr	48 hr	Diet (A)	Time (h)(B)	A x B
L-histidine ^*∗*^	7.8 ± 0.9	9.7 ± 0.1	8.8 ± 0.3	6.4 ± 0.3	13.9 ± 1.5	12.5 ± 1.5	6.7 ± 0.9	10.2 ± 0.9	13.0 ± 2.4	8.4 ± 2.4	13.4 ± 3.5	11.6 ± 1.4	6.7 ± 0.8	8.0 ± 1.1	9.9 ± 1.1	0.015	<0.0001	0.070
Hydroxy-L-Proline	3.6 ± 0.4	2.1 ± 0.1	2.0 ± 0.3	2.2 ± 0.1	2.3 ± 0.3	2.5 ± 0.5	4.1 ± 0.2	3.8 ± 0.3	4.7 ± 0.6	3.9 ± 1.3	3.4 ± 0.5	3.2 ± 0.2	2.0 ± 0.1	1.6 ± 0.1	1.7 ± 0.4	<0.0001	0.104	0.170
L-Arginine ^*∗*^	14.7 ± 1.5	16.7 ± 0.5	18.8 ± 3.3	12.3 ± 1.3	23.1 ± 2.4	24.4 ± 2.5	15.9 ± 1.3	23.9 ± 3.4	30.2 ± 4.5	16.6 ± 6.8	24.5 ± 1.5	21.1 ± 3.0	12.5 ± 1.0	16.2 ± 0.9	15.2 ± 2.4	0.0004	<0.0001	0.102
Ethanolamine	0.3 ± 0.02	0.2 ± 0.01	0.2 ± 0.02	0.4 ± 0.1	0.3 ± 0.04	0.3 ± 0.04	0.3 ± 0.01	0.2 ± 0.03	0.2 ± 0.05	0.5 ± 0.1	0.3 ± 0.03	0.4 ± 0.02	0.4 ± 0.05	0.2 ± 0.02	0.2 ± 0.04	<0.0001	<0.0001	0.915
L-Serine	16.8 ± 2.5	11.4 ± 0.2	11.6 ± 0.4	14.1 ± 1.3	15.1 ± 0.8	14.8 ± 2.5	15.1 ± 0.8	12.5 ± 0.4	16.1 ± 2.6	17.2 ± 3.5	15.2 ± 2.8	13.5 ± 0.7	14.2 ± 3.4	11.5 ± 1.1	14.0 ± 2.3	0.420	0.089	0.302
Glycine	19.4 ± 1.7	10.3 ± 0.5	10.1 ± 0.6	16.1 ± 0.9	12.7 ± 1.7	13.2 ± 1.8	18.8 ± 0.6	13.3 ± 0.9	15.9 ± 2.0	21.1 ± 4.8	14.0 ± 1.5	13.6 ± 0.6	14.9 ± 2.3	9.8 ± 0.7	11.5 ± 1.7	0.003	<0.0001	0.240
L-Aspartic acid	31.5 ± 3.0	20.1 ± 0.5	23.1 ± 1.5	26.2 ± 0.7	23.1 ± 0.4	23.2 ± 1.1	32.4 ± 1.4	24.0 ± 2.1	29.8 ± 1.6	35.1 ± 8.6	26.5 ± 1.1	25.4 ± 1.1	27.6 ± 2.2	24 ± 0.7	28.3 ± 0.1	0.017	<0.0001	0.145
Taurine ^*∗*^	1.2 ± 0.1	0.2 ± 0.1	0.3 ± 0.1	1.8 ± 1.1	0.2 ± 0.05	0.3 ± 0.05	2.0 ± 1.0	0.4 ± 0.4	0.3 ± 0.05	2.4 ± 1.0	0.5 ± 0.3	1.5 ± 1.7	4.3 ± 0.5	0.3 ± 0.01	0.4 ± 0.2	0.076	<0.0001	0.081
b-Alanine	0.2 ± 0.03	0.3 ± 0.01	0.3 ± 0.04	0.3 ± 0.04	0.3 ± 0.1	0.3 ± 0.01	0.3 ± 0.01	0.2 ± 0.03	0.2 ± 0.02	0.3 ± 0.1	0.2 ± 0.03	0.3 ± 0.01	0.3 ± 0.05	0.3 ± 0.1	0.3 ± 0.05	0.205	0.345	0.433
L-Threonine ^*∗*^	17.5 ± 1.5	10.5 ± 0.2	11 ± 0.5	14.3 ± 0.4	12.4 ± 1.4	12.7 ± 1.3	17.3 ± 0.8	11.9 ± 0.5	15.1 ± 1.7	19.4 ± 5.0	13.0 ± 0.8	12.8 ± 0.3	14.4 ± 1.7	10.6 ± 0.4	13.0 ± 1.5	0.077	<0.0001	0.197
L-Glutamic acid	49.0 ± 3.8	19.9 ± 1.2	22.5 ± 1.6	40.2 ± 1.4	23.3 ± 0.7	24.1 ± 1.0	49.2 ± 2.1	23.7 ± 2.2	29.4 ± 1.8	52.6 ± 12.6	25.7 ± 1.0	25.0 ± 0.6	44.3 ± 1.6	25.3 ± 0.2	29.3 ± 1.0	0.105	<0.0001	0.184
Citruline	0.2 ± 0.1	0.2 ± 0.1	0.2 ± 0.1	0.1 ± 0.03	0.2 ± 0.1	0.2 ± 0.1	0.1 ± 0.04	0.2 ± 0.02	0.1 ± 0.04	0.1 ± 0.01	0.5 ± 0.3	0.2 ± 0.05	0.1 ± 0.1	0.3 ± 0.2	0.1 ± 0.1	0.391	0.094	0.467
L-Alanine	18.4 ± 1.0	12.7 ± 0.1	14.1 ± 0.5	16.5 ± 0.2	16 ± 0.2	16.7 ± 0.7	18.4 ± 0.5	14.4 ± 1.2	18.5 ± 1.4	21.1 ± 5.2	16.9 ± 0.6	16.1 ± 0.5	16.1 ± 1.4	14.4 ± 0.7	16.4 ± 0.6	0.066	0.002	0.151
GABA	0.4 ± 0.01	0.1 ± 0.01	0.1 ± 0.02	0.4 ± 0.02	0.1 ± 0.01	0.1 ± 0.01	0.4 ± 0.01	0.1 ± 0.01	0.05 ± 0.01	0.4 ± 0.01	0.1 ± 0.02	0.05 ± 0.01	0.4 ± 0.01	0.1 ± 0.08	0.1 ± 0.01	0.690	<0.0001	0.954
L-Proline	13.8 ± 0.9	9.3 ± 0.3	10.0 ± 0.6	12.8 ± 0.3	11.5 ± 0.6	12.4 ± 1.0	13.4 ± 0.7	10.5 ± 0.8	12.8 ± 1.1	16.2 ± 4.0	11.9 ± 0.4	11.4 ± 0.1	13.4 ± 0.7	12.2 ± 0.3	14.0 ± 0.9	0.100	0.0002	0.101
delta-Hydroxylysine	0.9 ± 0.1	^−^	^−^	0.7 ± 0.1	^−^	^−^	1.0 ± 0.2	–	–	0.9 ± 0.2	–	–	0.5 ± 0.04	–	–	0.005	–	–
L-Ornithine	3.7 ± 1.9	1.1 ± 0.2	1.3 ± 0.5	3.2 ± 1.5	1.1 ± 0.3	1.2 ± 0.1	2.1 ± 0.8	0.8 ± 0.2	0.5 ± 0.1	2.0 ± 0.5	0.4 ± 0.2	0.4 ± 0.1	3.7 ± 2.3	0.2 ± 0.2	0.1 ± 0.04	0.341	<0.0001	0.831
L-Valine ^*∗*^	16.5 ± 1.3	11.0 ± 0.3	11.9 ± 0.5	14.2 ± 0.1	13.5 ± 0.8	13.7 ± 1.0	17.0 ± 0.8	12.1 ± 1.1	15.2 ± 1.3	20.5 ± 5.9	14.1 ± 1.0	14.2 ± 0.3	15.4 ± 1.0	13.0 ± 0.4	15.0 ± 1.0	0.094	0.0002	0.221
L-Methionine ^*∗*^	0.5 ± 0.03	6.9 ± 0.1	6.7 ± 0.3	0.4 ± 0.02	7.0 ± 0.8	7.3 ± 0.9	0.4 ± 0.02	6.3 ± 0.2	8.3 ± 1.2	0.5 ± 0.2	6.1 ± 0.7	6.0 ± 0.4	0.3 ± 0.02	5.5 ± 0.2	6.7 ± 0.9	0.071	<0.0001	0.117
L-Lysine ^*∗*^	23.1 ± 2.5	7.4 ± 0.2	9.0 ± 0.4	18.6 ± 0.2	7.6 ± 0.6	9.9 ± 1.0	24.7 ± 1.4	10.5 ± 1.7	9.5 ± 0.5	25.3 ± 5.5	7.3 ± 1.2	5.6 ± 0.7	16.7 ± 0.7	3.5 ± 0.9	2.4 ± 0.3	<0.0001	<0.0001	0.006
L-Anserine	1.6 ± 0.9	–	–	1.1 ± 0.1	–	–	1.2 ± 0.4	–	–	1.1 ± 0.2	–	–	2.1 ± 0.8	–	–	0.410	–	–
L-Cystine	1.4 ± 0.1	0.8 ± 0.1	0.9 ± 0.1	1.0	0.7 ± 0.1	0.7 ± 0.1	1.2 ± 0.1	0.6 ± 0.2	1.1 ± 0.5	1.3 ± 0.3	0.6 ± 0.1	0.7 ± 0.2	1.7 ± 0.1	0.5 ± 0.1	0.6 ± 0.2	0.362	<0.0001	0.073
L-Tyrosine	12.2 ± 0.6	8.0 ± 0.2	7.7 ± 0.7	11.5 ± 0.4	10.5 ± 1.6	10.9 ± 1.9	11.0 ± 0.5	7.6 ± 0.2	9.8 ± 1.6	15.8 ± 5.3	10.5 ± 1.8	10.5 ± 1.2	9.8 ± 0.9	8.0 ± 0.5	9.7 ± 2.0	0.036	0.003	0.467
L-Leucine ^*∗*^	22.2 ± 1.4	19.2 ± 1.1	20.9 ± 1.4	18.9 ± 0.6	23.0 ± 1.3	23.8 ± 1.7	21.3 ± 1.1	20.5 ± 1.7	25.9 ± 2.4	24.4 ± 6.3	23.2 ± 1.2	22.7 ± 0.5	20.6 ± 1.0	23.4 ± 0.5	28.0 ± 2.47	0.180	0.020	0.068
L-isoleucine ^*∗*^	13.7 ± 0.6	10.8 ± 0.5	11.8 ± 0.7	11.5 ± 0.2	12.5 ± 0.8	12.8 ± 1.0	14.5 ± 0.9	11.7 ± 1.2	14.7 ± 1.2	16.7 ± 4.9	13.3 ± 0.9	13.3 ± 0.3	13.0 ± 0.6	12.7 ± 0.3	14.9 ± 1.1	0.103	0.087	0.221
L-Phenylalanine ^*∗*^	16.4 ± 0.5	11.2 ± 0.2	10.3 ± 1.1	12.5 ± 0.3	11.9 ± 1.5	12.4 ± 1.8	15.0 ± 0.7	11 ± 0.5	13.8 ± 2.3	18 ± 5.9	12.3 ± 1.9	12.4 ± 1.2	14.9 ± 1.0	12.3 ± 0.5	14.7 ± 3.1	0.490	0.004	0.360
L-Tryptophan ^*∗*^	0.3 ± 0.1	0.1 ± 0.01	0.1 ± 0.02	0.2 ± 0.07	0.1 ± 0.05	0.1 ± 0.02	0.2 ± 0.04	0.1 ± 0.01	0.1 ± 0.02	0.1 ± 0.01	0.2 ± 0.1	0.1 ± 0.1	0.2 ± 0.1	0.1 ± 0.03	0.1 ± 0.01	0.709	0.001	0.462

*Note*: Data are represented by means of three replicates ± standard deviation (*n* = 3). For each parameter, significant differences are shown by different superscripts (Tukey's test, *p* < 0.05). Values with ( ^*∗*^) are essential amino acids in abalone. (−) Data not detectable. Abbreviations: Diet F (fish meal based), FI (fish meal + insect meal), FG (fish meal + grape marc), FIG (fish meal + insect meal + grape marc), and commercial feed (CF).

**Table 6:** Phosphorus (P) and carbon : nitrogen (C : N) leaching (tanks without abalone) of experimental diets and commercial feed.

	Diet																	
	F			FI			FG			FIG			CF			ANOVA (*p* value)		
Time in seawater (hr)	0 hr	24 hr	48 hr	0 hr	24 hr	48 hr	0 hr	24 hr	48 hr	0 hr	24 hr	48 hr	0 hr	24 hr	48 hr	Diet (A)	Time (h)(B)	A x B
Phosphorus ^*∗*^	1076.2 ± 99.8	1048.8 ± 35.2	1106.9 ± 15.9	786.0 ± 1.3	830.6 ± 22.4	823.9 ± 6.2	943.5 ± 13.4	804.2 ± 20.1	901.6 ± 34.0	666.5 ± 9.3	706.3 ± 23.4	745.0 ± 16.6	876.6 ± 8.7	1031.8 ± 90.7	988.4 ± 18.6	<0.001	0.058	0.003
C : N ratio	6.5 ± 0.1	7.8 ± 0.2	8.0 ± 0.2	8.6 ± 0.3	8.2 ± 0.1	7.9 ± 0.3	7.5 ± 0.2	7.2 ± 0.1	7.2 ± 0.1	8.2 ± 0.4	8.1 ± 0.6	7.7 ± 0.1	7.6 ± 0.1	8.6 ± 0.1	8.1 ± 0.04	<0.001	0.047	0.004

*Note*: Data represent means and standard deviation (*n* = 3). A significant level of *p* < 0.05 was used for all statistical tests. Abbreviations: Diet F (fish meal based), FI (fish meal + insect meal), FG (fish meal + grape marc), FIG (fish meal + insect meal + grape marc), and commercial feed (CF).  ^*∗*^Phosphorus in ppm per gram diet.

**Table 7:** Phosphorus and carbon : nitrogen leaching (C : N) (tanks with abalone) of experimental diets and commercial feed.

					Diet								
	F				FI				FG				
Time in seawater (hr)	0 hr	24 hr	48 hr	96 hr	0 hr	24 hr	48 hr	96 hr	0 hr	24 hr	48 hr	96 hr
Phosphorus ^*∗*^	1446.8 ± 60.5	2506.5 ± 33.2	2334.3 ± 87.0	2403.2 ± 53.0	908.9 ± 42.5	2022.6 ± 22.3	1940.8 ± 119.9	1891.5 ± 67.0	1110.5 ± 20.6	1217.4 ± 47.6	1220.7 ± 37.0	1192.9 ± 23.0
C : N ratio	6.5 ± 0.1	6.2 ± 0.2	6.0 ± 0.2	5.7 ± 0.2	8.6 ± 0.3	8.1 ± 0.2	7.9 ± 0.2	8.2 ± 0.2	7.5 ± 0.2	7.0 ± 0.1	6.8 ± 0.3	7.0 ± 0.9
	FIG				CF					ANOVA (*P* value)		
Time in seawater (hr)	0 hr	24 hr	48 hr	96 hr	0 hr	24 hr	48 hr	96 hr		Diet (A)	Time (h) (B)	A x B
Phosphorus ^*∗*^	466.2 ± 42.4	631.3 ± 30.4	676.4 ± 20.2	615.4 ± 41.7	795.4 ± 23.3	1740.6 ± 29.2	699.6 ± 83.5	666.2 ± 13.7		<0.0001	<0.0001	<0.0001
C : N ratio	8.2 ± 0.4	8.7 ± 0.7	8.2 ± 0.4	7.5 ± 0.2	7.6 ± 0.1	8.0 ± 0.03	7.5 ± 0.06	7.4 ± 0.02		<0.0001	0.003	0.290

*Note*: Data represent means and standard deviation (*n* = 3). A significant level of *p* < 0.05 was used for all statistical tests. Abbreviations: Diet F (fish meal based), FI (fish meal + insect meal), FG (fish meal + grape marc), FIG (fish meal + insect meal + grape marc), and commercial feed (CF).  ^*∗*^Phosphorus in ppm per gram diet.

**Table 8:** Growth performance and nutrient utilisation of *Haliotis iris* fed four different experimental diets and commercial feed.

	Diet				
	F	FI	FG	FIG	CF
Growth performance					
Daily weight gain (g)	0.027 ± 0.013	0.105 ± 0.096	0.025 ± 0.013	0.037 ± 0.022	0.016 ± 0.005
Daily Shell length gain (mm)	0.068 ± 0.042	0.054 ± 0.016	0.040 ± 0.005	0.049 ± 0.009	0.168 ± 0.021
Daily Shell width gain (mm)	0.020 ± 0.011	0.049 ± 0.013	0.040 ± 0.017	0.041 ± 0.014	0.050 ± 0.037
Feed conversion ratio	0.929 ± 0.417	0.770 ± 0.248	1.701 ± 1.372	1.172 ± 1.070	0.858 ± 0.216
*Enzyme activity*					
Trypsin (U mg protein^−1^)	0.008 ± 0.003^ab^	0.007 ± 0.001^b^	0.011 ± 0.004^a^	0.009 ± 0.004^ab^	0.007 ± 0.001^b^
Amylase (U mg protein^−1^)	4.014 ± 1.241^b^	5.013 ± 1.280^b^	18.015 ± 4.564^a^	14.009 ± 5.523^a^	13.787 ± 3.409^a^
Lipase (U mg protein^−1^)	0.013 ± 0.090	0.014 ± 0.011	0.010 ± 0.006	0.007 ± 0.004	0.006 ± 0.003
Protein in viscera (mg/g sample)	294.9 ± 55.4^b^	314.2 ± 29.1^ab^	313.0 ± 34.7^ab^	349.3 ± 29.6^ab^	366.8 ± 39.1^a^

*Note*: Data represent means and standard deviation (*n* = 45). For each parameter, significant differences are shown by different superscripts (Tukey's test, *p* < 0.05). Abbreviations: Diet F (fish meal based), FI (fish meal + insect meal), FG (fish meal + grape marc), FIG (fish meal + insect meal + grape marc), and commercial feed (CF).

## Data Availability

All the data in the article are available from the corresponding author upon reasonable request.
